# The recombination dynamics of *Staphylococcus aureus* inferred from *spA* gene

**DOI:** 10.1186/s12866-016-0757-9

**Published:** 2016-07-11

**Authors:** Célio D. Santos-Júnior, António Veríssimo, Joana Costa

**Affiliations:** Department of Molecular Biology and Evolutionary Genetics, Federal University of São Carlos (UFSCar), São Paulo, Brazil; CNC - Center for Neuroscience and Cell Biology, University of Coimbra - Rua Larga, Faculdade de Medicina, Pólo I, 1° andar, 3004-504 Coimbra, Portugal; Department of Life Sciences, University of Coimbra - Calçada Martim de Freitas, 3000-456 Coimbra, Portugal

**Keywords:** Staphylococcus aureus, Staphylococcal protein A, Recombination, Molecular evolution, *spA* typing, Virulence factor

## Abstract

**Background:**

Given the role of *spA* as a pivotal virulence factor decisive for *Staphylococcus aureus* ability to escape from innate and adaptive immune responses, one can consider it as an object subject to adaptive evolution and that variations in *spA* may uncover pathogenicity variations.

**Results:**

The population genetic structure was deduced from the extracellular domains of SpA gene sequence (domains A-E and the X-region) and compared to the MLST-analysis of 41 genetically diverse methicillin-resistant (MRSA) and methicillin-susceptible (MSSA) *S. aureus* strains. Incongruence between tree topologies was noticeable and in the inferred *spA* tree most MSSA isolates were clustered in a distinct group. Conversely, the distribution of strains according to their *spA*-type was not always congruent with the tree inferred from the complete *spA* gene foreseeing that *spA* is a mosaic gene composed of different segments exhibiting different evolutionary histories. Evidences of a network-like organization were identified through several conflicting phylogenetic signals and indeed several intragenic recombination events (within subdomains of the gene) were detected within and between CC’s of MRSA strains. The alignment of SpA sequences enabled the clustering of several isoforms as a result of non-randomly distributed amino acid variations, located in two clusters of polymorphic sites in domains D to B and Xr (a). Nevertheless, evidences of cluster specific structural arrangements were detected reflecting alterations on specific residues with potential impact on *S. aureus* pathogenicity.

**Conclusions:**

The detection of positive selection operating on *spA* combined with frequent non-synonymous mutations, domain duplication and frequent intragenic recombination events represent important mechanisms acting in the evolutionary adaptive mechanism promoting *spA* genetic plasticity. These findings argue that crucial allelic forms correlated with pathogenicity can be identified by sequences analysis enabling the design of more robust schemes.

**Electronic supplementary material:**

The online version of this article (doi:10.1186/s12866-016-0757-9) contains supplementary material, which is available to authorized users.

## Background

*Staphylococcus aureus* is recognized both as a widespread commensal organism on the human skin and anterior nose, as well as a notorious human pathogen in community-acquired and nosocomial infections, responsible for a wide range of diseases. *S. aureus* can asymptomatically colonize individuals, and indeed, approximately 30 % of humans are asymptomatic nasal carriers of this bacterium. These carriers are presumed to represent the initial mode of transmission of *S. aureus*, usually by direct contact, nevertheless contact with contaminated objects and surfaces has to be considered. Several host factors, like loss of the normal skin barrier, and underlying diseases predispose to infection [1, 2].

The ability of *S. aureus* to acquire resistance to antibiotic is widely known. In fact, the introduction of methicillin, a penicillinase-resistant penicillin, in the sixties contributed to the appearance of methicillin-resistance *S. aureus* (MRSA) [3] compromising the efficiency of most β-lactam antibiotics. Today, infections caused by MRSA reached epidemic proportions with significant human morbidity posing a major health problem worldwide [4]. The early MRSA clones were hospital-associated (HA-MRSA); however, during the last decade, community-associated MRSA (CA-MRSA) clones are globally distributed, both in the community and in healthcare facilities [5, 6]. Beyond the reported increase on the prevalence and incidence of these highly diverse CA-MRSA strains, they seem to be particularly virulent given the presence of manifold virulence-related factors [7, 8]. The abovementioned circumstances are exacerbated by the absence of a protective vaccine and by the fact that *S. aureus* infection in humans does not induce protective immunity. This phenomenon involves the unique immune globulin G-binding protein A, or staphylococcal protein A (SpA), a critical virulence factor that allows *S. aureus* to avoid innate and adaptive immune responses [9–11].

SpA is a surface molecule that binds to Fcγ of human and animal immunoglobulin (Ig), a defense mechanism that hinders the capacity of antibodies with specific binding activities for the *S. aureus* surface to enable Fc receptor-mediated opsonophagocytosis and bacterial killing [12]. The SpA precursor has a N-terminal signal peptide (YSIRK pfam 04650) and a sorting signal in the C-terminal for covalent anchoring to the cell wall (LysM pfam 01476) [13]. The mature SpA comprises in the N-terminal four to five 56–61 residue Ig binding domains, A to E respectively, that fold into triple helical packs linked by short connectors [14, 15]. This Ig-binding region is followed by the variable length region X, that comprises Xr, a variable number (from 3 to 15) of tandemly repeated 24-bp units, and Xc, a domain with a uncommon sequence that restricts the cell wall anchor structure of SpA [16, 17]. The Fcγ domain of IgG, as well as the Fab domain of V_H_3 class IgG and IgM, are captured by the five immunoglobulin-binding domains (IgBDs) of SpA preventing staphylococci opsonophagocytic killing. Moreover, B cell superantigen activity is triggered by SpA through cross-linking of V_H_3 type B cell receptors (surface IgM), resulting in supraclonal expansion as well as apoptotic collapse of the activated B cells, indicating that antibodies production and B cells function have a fundamental role in *S. aureus* infections [9–11, 14, 18–20].

Due to the significant human morbidity caused by this bacterium different typing methods, particularly molecular techniques, have been developed for epidemiological tracing and population genetic studies. Frénay and Colleagues [21] developed a fast, discriminatory and reliable method for *S. aureus* epidemiological studies based on the sequence variation of the polymorphic region X of the *spA* locus [22]. This allows a rapidly characterization of the isolates through comparison of SpA sequence with Ridom SpaServer database [23] in which different strains are assigned to distinct *spA* types according to the generated profile. Moreover, cluster analysis is then possible through the algorithm based on repeat pattern (BURP) implemented into StaphType [24]. Indeed, *S. aureus* strains assigned as more virulent were found to have more than seven repeat units within the X region. Such a correlation presumes that the longer X region is, more precise and stronger is the binding of encoded SpA to Fc fragment of IgG, resulting in a more effective defense against host immunological system [25, 26].

The discriminatory power of *spA* typing is inferior to that of Pulsed-field gel electrophoresis (PFGE), but the clusters identified by *spA* typing and Multilocus sequence typing (MLST) correlate well at the level of clonal complexes, so that clonal assignment is reliable *S. aureus* surveillance is nowadays mostly decentralized since *spA* typing is a highly reproducible and portable method, replacing PFGE in many reference laboratories [27, 28].

Given the role of SpA as critical virulence factor that allows *S. aureus* to escape innate and adaptive immune responses, it is foreseeable that host specialization and clonal expansion through adaptive evolution may target this gene product and that changes in *spA* may display an increase in *S. aureus* pathogenicity. Our goal was to assess the population genetic structure of *S. aureus* deduced from *spA* gene and to determine the molecular mechanisms driving the evolution of this virulence-related factor. The study of the genetic diversity and distribution of MRSA and MSSA isolates is important to assessment the population genetic structure and inference of phylogenetic relationships. Likewise, an in depth comparison may help to determine what percentage of emerging MRSA strains are linked with single *spA* sequences, and, accordingly, may indeed be identified based on *spA* typing. For this purpose we used the complete gene sequence from the extracellular domains, and not just the hypervariable region X, since the Ig binding domains also play a crucial role in *S. aureus* pathogenicity [9–11], from 41 epidemiologically unrelated MRSA and MSSA genetically diverse strains of *S. aureus*.

Our results argue that intragenic recombination is an important strategy in the evolutionary adaptive process fostering *spA* genetic plasticity. Furthermore, all MSSA strains were clustered in a single discrete group reinforcing the use of SpA as a discriminative gene.

## Methods

### *spA* and MLST allelic profiling, clustering and phylogenetic analysis

The entire genome sequence of 41 *Staphylococcus aureus* strains (Table 1) was used to retrieve the extracellular domains of the virulence factor SpA responsible for the ability of *S. aureus* to escape innate and adaptive immune responses [9–11]. The YSIRK_signal (pfam 04650), LysM (pfam01476) and anchoring motifs were trimmed for each *spA* coding region, leaving the extracellular portion of SpA, corresponding to protein domains A-E plus the X region comprising the octapeptide repeat 2–1 to 2–10 domain, previously classified by [14, 29] and available at UniprotKB with the entry P38507.

**Table 1:** *S. aureus* strains used in this study

Strain	MLST		*spa* type (Ridom)	Spa repeat pattern	MSSA/MRSA	Genome
	ST	CC				Accession
JH1	105	5	t002	26-23-17-34-17-20-17-12-17-16	MRSA	NC_009632
JH9	105	5	t002	26-23-17-34-17-20-17-12-17-16	MRSA	NC_009487
Mu3	5	5	t002	26-23-17-34-17-20-17-12-17-16	MRSA	NC_009782
Mu50	5	5	t002	26-23-17-34-17-20-17-12-17-16	MRSA	NC_002758
N315	5	5	t002	26-23-17-34-17-20-17-12-17-16	MRSA	NC_002745
ECT-R2	5	5	t002	26-23-17-34-17-20-17-12-17-16	MSSA	NC_017343
ED98	5	5	t002	26-23-17-34-17-20-17-12-17-16	MSSA	NC_013450
04-02981	225	5	t003	26-17-20-17-12-17-17-16	MRSA	NC_017340
COL	250	8	t008	11-19-12-21-17-34-24-34-22-25	MRSA	CP000046.1
FPR3757	8	8	t008	11-19-12-21-17-34-24-34-22-25	MRSA	NC_007793
Newman	8	8	t008	11-19-12-21-17-34-24-34-22-25	MSSA	NC_009641
ST398	398	15	t011	8-16-2-25-34-24-25	MRSA	NC_017333
MRSA252	36	30	t018	15-12-16-2-16-2-25-17-24-24-24	MRSA	NC_002952
TCH60	NI	NI	t019	8-16-2-16-2-25-17-24	MSSA	NC_017342
M1	8	8	t024	11-12-21-17-34-24-34-22-25	MRSA	NC_021059
T0131	239	8	t030	15-12-16-2-24-24	MRSA	NC_017347
08BA02176	398	15	t034	8-16-2-25-2-25-34-24-25	MRSA	NC_018608
JKD6008	239	8	t037	15-12-16-2-25-17-24	MRSA	NC_017341
TW20	239	8	t037	15-12-16-2-25-17-24	MRSA	NC_017331
11819-97	80	80	t044	7-23-12-34-34-33-34	MRSA	NC_017351
10388	228	5	t1003	26-17-20-17-34-17-20-17-12-17-16	MRSA	HE579059.1
10497	228	5	t1003	26-17-20-17-34-17-20-17-12-17-16	MRSA	HE579061.1
15532	228	5	t1003	26-17-20-17-34-17-20-17-12-17-16	MRSA	HE579063.1
16035	228	5	t1003	26-17-20-17-34-17-20-17-12-17-16	MRSA	HE579065.1
16125	228	5	t1003	26-17-20-17-34-17-20-17-12-17-16	MRSA	HE579067.1
18341	228	5	t1003	26-17-20-17-34-17-20-17-12-17-16	MRSA	HE579069.1
18412	228	5	t1003	26-17-20-17-34-17-20-17-12-17-16	MRSA	HE579071.1
18583	228	5	t1003	26-17-20-17-34-17-20-17-12-17-16	MRSA	HE579073.1
HO50960412	22	22	t1041	26-23-23-13-23-31-29-17-28	MRSA	NC_017763
MW2	1	1	t128	7-23-23-21-16-34-33-13	MRSA	NC_003923
JKD6159	93	93	t202	11-17-23-17-17-16-16-25	MRSA	NC_017338
VC40	8	8	t211	11-19-12-12-21-17-34-24-34-22-25	MRSA	NC_016912
NCTC8325	8	8	t211	11-19-12-12-21-17-34-24-34-22-25	MSSA	NC_007795
133	842	97	t2678	3-16-12-21-17-23-13-17-17-17-23-24	MSSA	NC_017337
M013	59	59	t437	4-20-17-20-17-25-34	MRSA	NC_016928
RF122	151	705	t529	4-34	MSSA	AJ938182.1
71193	398	15	t571	8-16-2-25-2-25-34-25	MSSA	NC_017673
476	1	1	t607	7-16-23-21-16-34-33-13	MSSA	NC_002953
TCH1516	8	8	t622	11-19-12-21-17-34-22-25	MSSA	NC_010079
LGA251	425	425	t6300	14-44-12-17-23-18-110-17-17-23-24	MRSA	NC_017349
MSHR1132	1850	75	NI	259-31-17-17-17-22-17-17-23-17-22	MRSA	NC_016941

*MLST* multilocus sequence typing; *ST* sequence type; *CC* clonal complex; *MRSA* methicillin-resistance *S. aureus*; *MSSA* methicillin-susceptible *S. aureus*

The X region from *spA* alleles,, composed by a series of repeats of 21 to 27 bp, was retrieved and submitted to DNAGear - The Spa Typing software that identifies *spA* alleles, detects new repeats and new *spA* types and synchronizes automatically the results with the open access databases [30]. *spA* types were clustered into *spa*-CCs with the algorithm Based Upon Repeat Pattern (BURP) [24] with a distance cost of ≤5; Only *spA* types with more than four repeats were considered. Minimum spanning trees (MSTs) for *spA* data were calculated using Prim’s algorithm [31] with BURST clustering using the PubMLST website (http://pubmlst.org/). Moreover, entire genome sequence of the abovementioned *S. aureus* strains (Table 1) were used to retrieve the sequences from the 6 loci used for *S. aureus* Multi Locus Sequence Type (MLST) typing, namely, *arcc*, *aroe*, *glpf*, *gmk*, *pta*, *tpi*, *yqil*, using the Center for Genomic Epidemiology (CGE) server [32]. Alleles assignment was performed in accordance with the *S. aureus* MLST database and presented as an ordered numerical vector [33]. STs were clustered into CCs with eBURST v3 [34]. The identified CCs included two or more STs that differed in a single locus (single-locus variants) or two loci (double locus variants) and singletons were set as sequence types that didn’t group into a CC [34, 35].

### *spA* sequence analysis

The *spA* gene sequences from *S. aureus* strains (Table I) were used for phylogenetic analyses with MEGA5 package [36]. Alignment was performed with CLUSTAL software [37], included on MEGA5 package. The *spA* coding locus alignment was performed with the amino acid sequences with ClustalΩ [38], manually rectified if required. MEGA5 package was used to derive the multiple alignments of nucleotide and positions of doubtful homology were removed using Gblocks [39].

Maximum likelihood (ML) phylogenetic trees were constructed with PhyML 3.0 [40] for *spA* locus with JC model [41] determined by TOPALi V2.5 [42] and by jModeltest [43], using Akaike Information Criterion (AIC) [44, 45] and from amino acid alignment using JTT + G + F model [44] assessed by ProtTest 2.4 [46]. Supports for the nodes were evaluated by bootstrapping with 1000 pseudoreplicates.

For the SpA protein phylogeny, *spA* coding locus alignment was performed with the amino acid sequences using ClustalΩ [38], manually corrected when necessary.

DnaSP software [47] was used to perform the genetic variability analyses.

PSFIND and HAPPLOT written by Dr Thomas S. Whittam and available at the STEC Center website (http://www.shigatox.net/stec/cgi-bin/programs) were used to determine and graphically display the location of variable nucleotide positions

### Molecular Evolution

Neighbor-net analysis was performed and converted to a splits graph by SplitsTree4 software – version 4.6 [48, 49], as previously described [50]. Intragenic recombination was screened within the aligned sequences with GARD method [51] available in Datamonkey server [52] as previously described [53]. GARD results were confirmed [54] using a recombination cost “delta dirac” and mutation cost “Hamming” implemented in the Recco program [55].

RDP3 program [56] was performed to validate the obtained results [53] with the requirement that each potential event had to be detected simultaneously by three or more methods.

### Neutrality tests and positive selection analysis of *spA* gene

Tajima’s D [57], Fu and Li’s D* and F* [58] statistics were calculated [59] for testing the mutation neutrality hypothesis [60], with the program DNASP4.0 [47]. Estimates of the number of non-synonymous and synonymous substitutions at each locus (*dN*/*dS*) were calculated using the modified Nei–Gojobori method [61] with Jukes-Cantor correction [41] implemented in MEGA5 package [36].

Selecton version 2.1 software [62] was used to estimate the existence of positive and purifying selection at each amino acid site as previously described [50] from nucleotide sequences alignment constructed using the MEGA5 package [36]. A Likelihood Ratio Test (LRT) was run to assess the significance of the results by comparing two nested models: a null model that assumes no selection (M8a) [63] and an alternative model that does (M8) [64].

### Computational comparison of biochemical properties of different *SpA* isoforms

Representative sequences of each *spA* phylogenetic group were translated with standard genetic code with MEGA5 package [36]. The Raptor X server was used to model the corresponding translated sequences with the automated mode with refinement of structure and secondary structure prediction [65] which was used to FirstGlance viewing. The pI, Mw and the main characteristics (instability index - II, grand average of hydropathicity - GRAVY and aliphatic index - AI) were inferred with Compute pI/Mw tool and ProtParam tool, respectively, both available at SIB Bioinformatics Resource Portal [66]. The Protein Variability Server was used to determine the sequence variability within SpA isoforms using several variability metrics, namely Shannon Entropy, Simpson Diversity Index and Wu-Kabat Variability coefficient [67].

## Results

### Sequence analysis of *spA* gene

The extracellular domains of the virulence factor SpA responsible for the capability of *S. aureus* to escape innate and adaptive immune responses [9–11] were studied from 41 *S. aureus* strains (Table 1) in order to identify the mechanisms operating on the evolution of this crucial gene. All the studied MRSA and MSSA strains encoded the *spA* gene. The strains were selected since they represent the observed diversity within the *S. aureus* genome-sequenced strains available in NCBI (National Center for Biotechnology Information) and KEGG (Kyoto Encyclopedia of Genes and Genomes).

After performing the alignment of the gene sequences and the corresponding translation, several stop codons were identified, namely in strains ED98 (MSSA), HO50960412 (MRSA) and RF122 (MSSA). In strain HO 50960412 (MRSA) the nonsense mutation was due to an insertion in nucleotide number 664. Point mutations at nucleotides 499 and 943 in the SpA coding sequences from strains ED98 and RF122, respectively, lead to the insertion of translational stop codons (GAA - > TAA). These truncations took place upstream of the cell wall-binding recognition sequence LPXTG, indicating that the protein would be unable to bind to the cell wall, but instead secreted into the medium [17]. Additionally, the SpA-encoding sequence from ED98 (MSSA) and HO50960412 (MRSA) strains only displayed three complete Ig-binding domains, with an incomplete B-domain and an absent C- domain [14, 18]. The deletions of these domains were in frame not affecting the repeat region. SpA is highly conserved and isolates of *S. aureus* lacking this virulence factor have been rarely identified. Nevertheless, sporadically naturally occurring mutants have been observed that secreted SpA into the extracellular environment foreseeing that SpA bond to the cell wall may not be essential for the survival and virulence of *S. aureus* in the host [68]. Moreover, most of the Ig-binding region was intact in ED98 (MSSA) and HO50960412 (MRSA) strains, probably allowing the binding of SpA to the Fc region of IgG and to the Fab region of the V_H_3 subclass immunoglobulins, thus resulting in B lymphocyte apoptosis. Indeed, *S. aureus* strains with truncated SpA have been recently isolated from bacteraemia, infection and among carriers [68]. These strains were excluded from posterior analysis.

### *S. aureus* phylogeny inferred from *spA* sequences

**Fig. 1:**
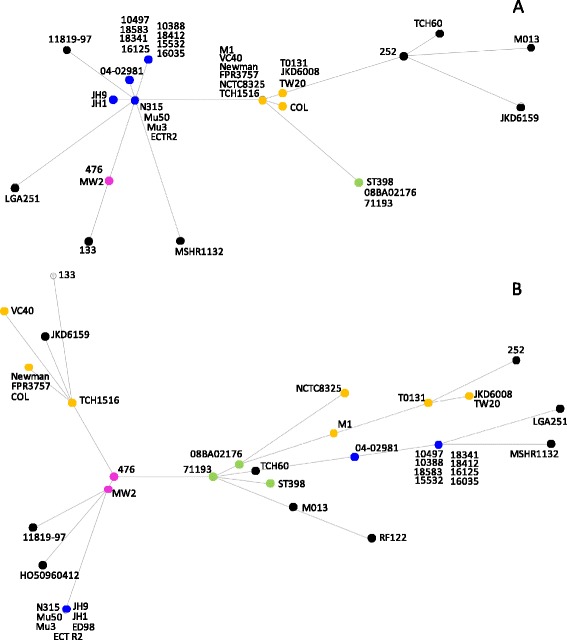
Population snapshot of *S. aureus* strains after **a** MLST BURTS clustering and **b** *spA* BURP grouping. The MLST minimum-spanning tree was obtained with BURST clustering.. *spA* types were clustered into *sp*A-CCs with the algorithm BURP. Strains are represented by circles highlighted according to their MLST-based clonal complexes, CC8 (yellow circles), CC15 (green circles), CC1 (purple circles) and CC5 (blue circles). Black circles represent singletons

Sixteen different Sequence Type (STs) were identified from the 38 *S. aureus* strains by comparison with the MLST database, and a new MLST profile was identified for the TCH60 strain (90-2-2-2-6-3-2) (Table 1). Most strains belonged to ST228, comprising 21 % of all strains (8 out of 38 strains); ST8 (15.8 %) and ST5 (10.5 %), all well-known epidemic types [69–71]. The 16 STs were split by eBURST into 2 main clonal complex (CC) (CC5 and −8), 2 minor CC’s (CC1 and −15), and 8 singletons (S30, −59, −75, −80, −93, −97, −425 and the new ST from strain MSHR1132) (Fig. 1a and Table 1). The major CC’s, CC5 and −8, comprised 4 and 3 different STs that included 15 and 10 *S. aureus* strains, respectively.

Twenty-two unique *spA* types were assigned based on the X region using the default settings of DNAGear (Table 1). We detected in strain MSHR1132 a combination of repeats at *spA* region X (259-31-17-17-17-22-17-17-23-17-22) not yet described in the *SpA* Ridom Server. The dominant *spA* type was t103 (*n* = 8, 21 %), followed by *spA* type t211 (*n* = 3, 8 %). s*pA* types were clustered using the BURP algorithm and the results were displayed as a MST (Fig. 1b). Comparisons between the two MSTs revealed that the clustering by *spA* typing was distinct from the clustering by MLST. Indeed, *spA* types disrupted the clonality determined by MLST, mostly evident for CC8 (Fig. 1b, highlighted in yellow).

**Fig. 2:**
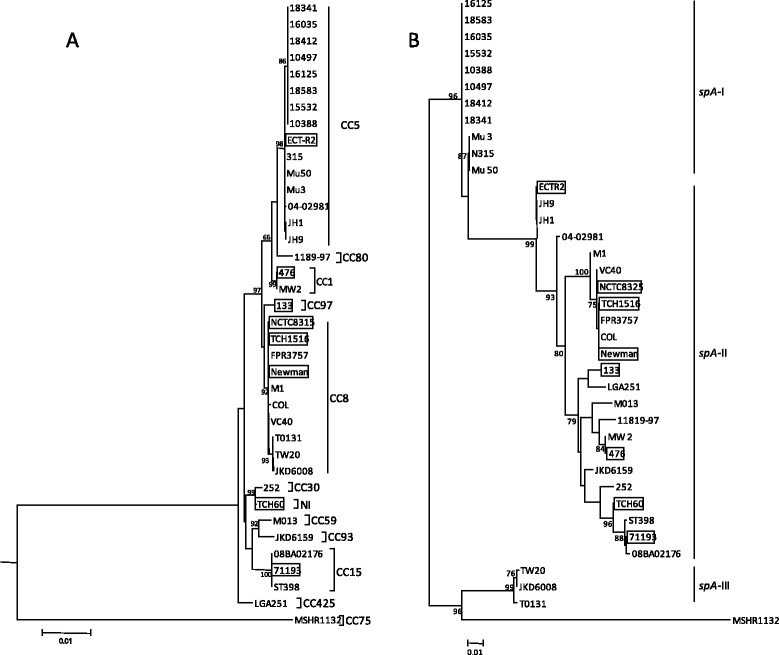
Molecular phylogenetic analysis by maximum likelihood method of *S. aureus* strains from **a** MLST concatenated genes and **b** *spA* gene. Bootstrap support values (1,000 replicates) for nodes higher than 50 % are indicated next to the corresponding node. Scale bar, 1 inferred amino acid substitutions per 100 nucleotides. CC’s and *spA* clusters are indicated next to corresponding strain. MSSA strains are boxed

In order to identify the mechanisms underlying *spA* molecular gene evolution, ML phylogenetic trees were obtained from the alignment of extracellular domains of *spA* locus and, for comparison purposes, from the MLST-concatenated alignment (Fig. 2). The MLST-concatenated inferred ML tree was in accordance with previously obtained eBURST analysis since each CC tends to cluster together (Fig. 2a). Conversely, the distribution of strains according to their *spA*-type was not always congruent with the topology of ML tree inferred from the *spA* sequences (Fig. 2b). Namely, strains Mu50, N315 and Mu, and strains ECTR2, JH1 and JH9, identified as *spA*-t002, were split into distinct clusters, respectively. While the Ridom SpaServer database [23] assigns *spA* sequences to distinct *spA* types according to variation in the tandem repeat region X from *spA*, the ML tree was inferred from complete extracellular domains of *spA* sequence. All other *S. aureus* strains that shared the same *spA*-type tend to cluster together and were distinct from all other groups (Fig. 2b).

The incongruent topology inferred from MLST and *spA* gene analysis (Fig. 2a and b, respectively) was evidenced by different branch sorting between the two trees. While in Fig. 2a most strains clustered in one group (97.56 %), in Fig. 2b, *S. aureus* strains were splitted into three discrete clusters supported by high bootstrap values. Furthermore, strains were not evenly distributed in these clusters. This incongruence’s are explained below in the context of recombination. When the *spA* sequence was analyzed, all the MSSA strains were grouped in a single cluster, in accordance with previous reports [21].

### Genetic variability of *spA* gene

Standard genetic diversity parameters, not dependent on sample size, were estimated based on *spA* and MLST-related loci to determine nucleotide diversity (Table 2). The average number of pairwise nucleotide differences (*k*), the overall haplotype diversity (*Hd*) and nucleotide diversity (π) for the 38 *spA* sequences were 44.570, 0.939 ± 0.025 and 0.0370 ± 0.0044, respectively. A particular analysis of π, with a sliding window plot (window length 100 bp, step size 25 bp), revealed diversity ranged from 0.003 to 0.034. Nucleotide diversity was higher between nucleotide 350–470 (within domain D), 680–810 (last portion of domain A and the entire domain B) and 960–1080 (domain Xr (a)), whereas the most conserved region was identified between nucleotide 840–960 (the entire domain C) (Additional file 1: Figure S1). These variable regions are discussed below in the context of amino acid substitutions.

**Table 2:** Summary of genetic diversity parameters for *spA* sequences and concatenate MLST loci from *S. aureus* strains

	*spA*	MLST
Sequence, *n*	38	38
Sequence length, *L*	1575	3186
Haplotypes, *h*	**21**	19
Haplotype diversity, *Hd* (standard deviation)	**0.939**	0.926
	(0.025)	(0.022)
Nucleotide diversity, π (standard deviation)	**0.0370**	0.0110
	(0.0044)	(0.0043)
Polymorphic sites, *S* (%)	191 (**12.13**)	**381** (11.96)
θ (from S) (standard deviation)	**0.03779**	0.02796
	(0.01126)	(0.00143)
Pairwise differences, *k*	**44.570**	35.090
Total number of mutations, η	184	**286**
Synonymous mutations (%)	133 (72.28)	297 (**76.74**)
Non-synonymous mutations (%)	51 (**27.72**)	90 (23.26)
*dN*/*dS*	**0.1348**	0.0970
*D* (*Tajima*)	−0.40 (*p* > 0.1)	-
D*	−1.34 (*p* > 0.1)	-
F*	−1.12 (*p* > 0.1)	-

Bold text was used to emphasize the higher value obtained between spA and MLST data

Analysis and comparison of *spA* at the nucleotide level showed mutations at 184 positions among *S. aureus* strains. One hundred and thirty three of those mutations were synonymous while 51 were nonsynonymous. The ratio between rate of non-synonymous substitutions (*dN*) to rate of synonymous substitutions (*dS*) was determined as an indicator of selective pressure acting on a protein-coding gene. The low *dN*/*dS* ratio obtained denoted that purifying (negative) selection has operated on theses alleles (Table 2), once variations are allowed providing that they do not result on significant disadvantage on any surviving variant. Tests to detect departure from neutrality, like D, D* and F* values, were non-significant suggesting that the null hypothesis of neutrality could not be rejected (Table 2). Therefore the pattern of variability observed in *spA* gene can be explained by the neutral process [57, 58, 72].

SpA had an average length of 361 amino acids with a standard deviation of 29 amino acids and a molecular weight average of 39.92 kDa with a standard deviation of 3.25 kDa. SpA revealed high polymorphism at amino acid level, transversally to all strains (Additional file 2: Figure S2). Among the 78 polymorphic sites, 74 were monomorphic mutations and 5 were dimorphic mutation [137 (A/N), 270 (A/D), 323 (A/G), 324 (Q/N), 387 (G/D)]. Nineteen different haplotypes were identified based on the amino acid sequences, with haplotype containing *spA* type t1003 having the highest frequency (8/38).

Phylogenetic tree analysis evidenced that most *spA* nucleotide polymorphisms resulted in amino acid changes since clusters inferred from deduced amino acid sequences of *spA* were consistent with the previously obtained nucleotide-based subgroups (Additional file 3: Figure S3). Indeed, we found 21 haplotypes which translate to 19 different protein sequences. Similar diversity parameters were found between *spA* and MLST loci (Table 2).

**Fig. 3:**
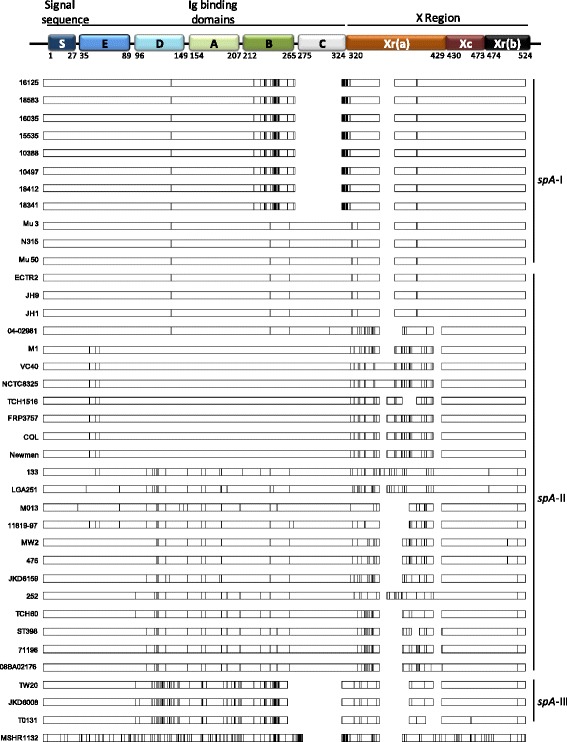
Graphical display of the location of polymorphic sites (SPNs and INDELs) of *spA* from *S. aureus* strains using the program HAPPLOT when aligned with *S. aureus* strain 18583. Polymorphic nucleotide sites based upon pairwise comparisons are represented by vertical lines

In order to find evidences for the existence of recombination events, namely the presence of mosaic patterns within *spA* sequences, the Happlot program was used to visualize relative position between alleles and a guiding sequence. The previously defined *spA* clusters matched the readily identified clusters of polymorphic sites, as shown in Fig. 3. Sequences resembled within clusters and were different from those found in other clusters, clearly indicating the existence of SpA isoforms. Indeed, *spA*-II cluster denoted a remarkable degree of both nucleotide and amino acid polymorphism.

### Reticulate evolutionary events inferred from *spA* sequences

**Fig. 4:**
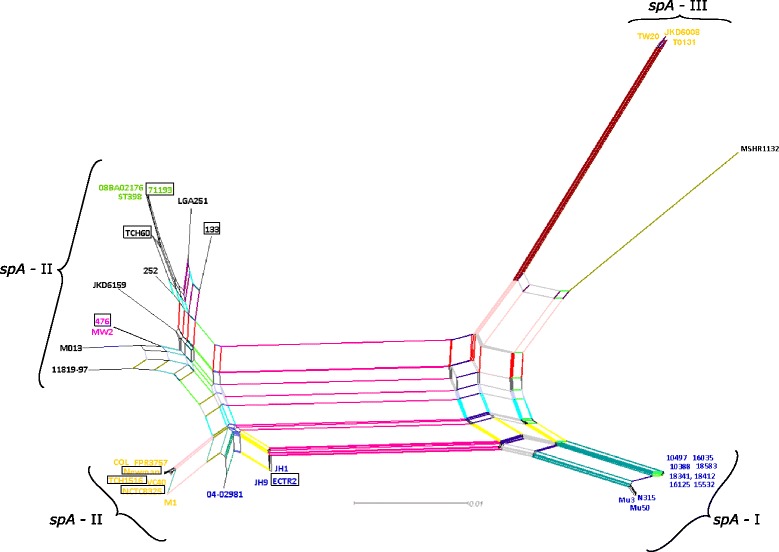
Neighbor-net phylogenetic network showing the relationships among *S. aureus* strains. The split graph was estimated with SplitsTree4 from *p*-distances of the *spA* sequence alignment based on the Jukes–Cantor method. Strains highlighted according to their MLST-based CC’s (Table 1 and Fig. 1), Color code: CC8 (yellow circles), CC15 (green circles), CC1 (purple circles) and CC5 (blue circles). The relations between and within strains are illustrated by weighted splits with different colors representing simultaneously both grouping in the data and evolutionary distances between taxa, highlighting conflicting signals or alternative phylogenetic histories (recombination or gene transfer) in *spA* molecular evolution. MSSA strains are boxed

In order to determine the effect of recombination and horizontal gene transfer events into the phylogenetic relationships of *S. aureus* strains a Neighbor-Net analysis (Fig. 4) has been constructed. Evidences of a network-like evolution were clear, indicating lack of tree-like relationship between *spA* sequences. Nevertheless, it is still possible to reconstruct the previously defined groups from the ML phylogenetic analysis (Fig. 2b). The clusters previously identified were quite robust, presenting a complex diversifying history. Moreover, the divergence of clusters *spA*-I and *spA*-III from cluster *spA*-II, only group with MSSA strains, was noticeable (Fig. 4).

### Determining the influence of recombination in *spA* molecular evolution

**Fig. 5:**
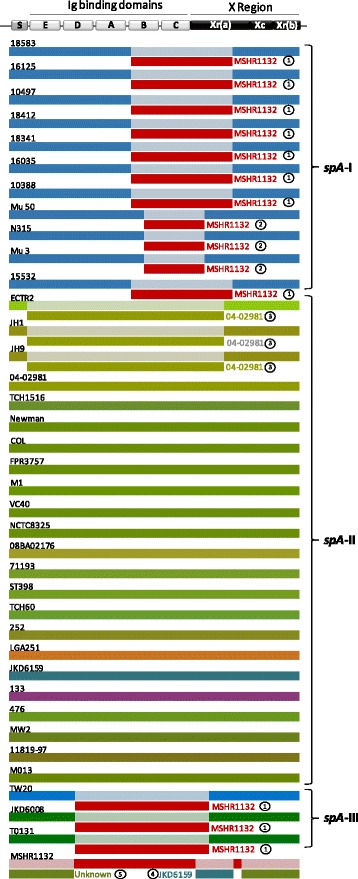
Unique recombination events detected on *spA* alignment. Each sequence is represented by a color and the recombination is evidenced by donor and is mapped onto the corresponding breaking point positions in the alignment. All analyses were evaluated with RDP and the most significant P value to support the findings are shown at Additional file 4: Table S1

The abovementioned results corroborate the occurrence of recombination events between and within distinct *spA* clusters. Indeed, evidences of individual recombination events were detected by two distinct approaches. Namely, GARD found evidences with statistical significance (*p* < 0.001, KH test) for at least 5 breaking-points, corroborated by Recco analysis from 1000 bootstraps. RDP analysis showed the same breaking-points with at least three different algorithms that were mapped into the corresponding ML phylogenetic tree (Fig. 5 and Additional file 4: Table S1).

This approach clarified the origin of several conflicting phylogenetic signals previously observed both in the ML and Neighbor-Net analysis since they were the result of Potential Recombination Events (PREs) (Fig. 2b and Fig. 4). The identified PREs were limited to MRSA strains with only one exception, the MSSA strain ECTR2, resolving the abovementioned complex evolutionary history of *spA* (Fig. 5). Namely, PRE1 involving eight of the strains clustered in *spA*-I and cluster *spA*-II with the ancestor MSHR1132 as minor parent, responsible for the bifurcation denoted in the ML and Neighbor-Net analysis (Fig. 2a and Fig. 4). Moreover, it was possible to identify PREs involving strains ECTR2, JH1 and JH9 with the ancestor 04–02981 as minor parent; and MSHR1132 that reconstructs previously assigned conflicting signals in the network, namely PRE’s number 3, 4 and 5 respectively (Fig. 5 and Additional file 4: Table S1).

### Forces operating in *SpA* evolution

Several neutrality testes previously described in Table 2 were employed to avoid the influence of positive selection on the accurate detection of recombination events [73]. In fact, variations on *spA* gene could be solely explained by the neutral hypothesis of evolution [57, 60, 58].

To further confirm this assumption the Selecton package [62] was used to screen the *spA* alignment for evidences of positive selection through a codon based ML method. The LRT strongly rejected the null hypothesis (*p* < 0.001) indicating that positive selection may have taken place (Additional file 5: Table S2). To restrict the effect that recombination could have on those tests by generating misleading results, the previously identified breakpoints by GARD were used to create the corresponding partitions that were subsequently individually submitted to Selecton. The LRT strongly rejected the null hypothesis revealing that positive selection may be operating within in the partition of SpA comprising the X region (partition 4). Then again no evidences of positive selection in partitions 1 to 3 were sought by the LRT test (Additional file 5: Table S2).

**Fig. 6:**
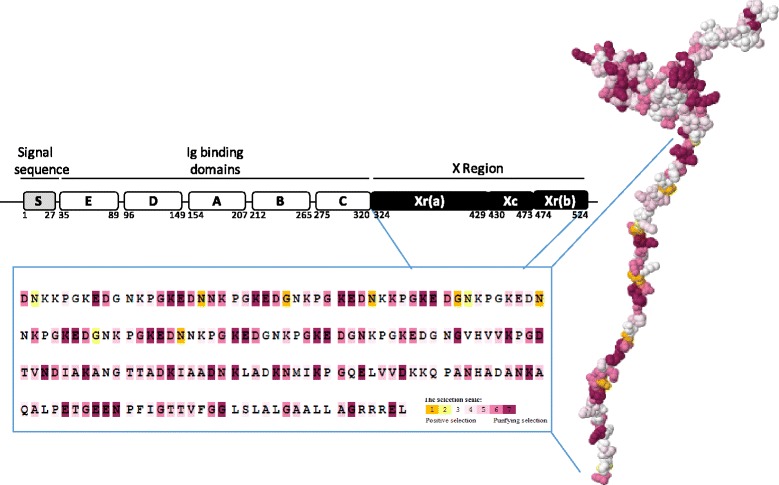
Estimates of both positive and purifying selection at each amino acid site of SpA calculated from the ratio of non-synonymous (Ka) to synonymous substitutions (Ks) [62]. Graphical display of selecton results with FirstGlance in Jmol where the Ka/Ks scores are colored-coded. Significant positive and purifying sites (*P*-value < 0.05) are colored in orange (color number 1) and magenta (color number 4), respectively

Since the previously performed LRTs indicated the presence of positive selection in *spA*, an empirical Bayesian analysis was performed to determine the posterior probability for each codon site to be under positive selection. For that, each partition was individually submitted to Selecton to identify the codons under positive selection. The Ka/Ks ratio was used to estimate both positive and purifying selection at each amino-acid site [74, 75]. The result for each codon was translated into a color scale graphically depicted on Fig. 6. Analyzing the obtained results one can determine that not a single residue was found to be under positive selection within the SpA Ig binding domains and signal sequence, anticipating that these SpA domains are under a strong negative constraint. However, several red and pink-colored sites were present in the partitions of SpA comprising the X region, representing positively selected codons with high statistical significance (Fig. 6).

### Biochemical comparison of SpA isoforms

The characteristics of SpA isoforms were evaluated and the distribution of Instability Index (II), Grand Average of Hydropathy (GRAVY) and Aliphatic Index (AI) followed the normal distribution (P_KS test_ > 0.05) (Table 3). The II measures provide an estimate of the protein stability, and II values smaller than 40 are predicted as stable [76]. Despite all the calculated values being higher than 40, this index presented a significant positive correlation with SpA clusters (r = 0.752, p = 7.89x10^−8^). The cluster with the lowest II was SpA-I (56.63 ± 0.13), while all the other clusters present an average under 59. These values estimate a potential instability for SpA proteins, common to all clusters, possibly explained by the existence of a membrane-dependent folding process in which final SpA conformations is achieved through hydrophobic interactions with phospholipids heads like previously described by Dowan and Bogdanov [77]. The AI of a protein is defined as the relative volume occupied by aliphatic side chains [78]. The AI was positively correlated with statistical significance with SpA clusters (r = 0.748, p = 1.03x10^−7^). The cluster SpA-I had an AI of 48.098 ± 0.56 while the others started at 53, showing an increasing of thermo-stability. The GRAVY [79] values were positively correlated with SpA clusters (r = 0.734, p = 7.89x10^−8^), similarly to II and AI. The higher values were obtained for SpA-III (−1.346 ± 0.018), demonstrating that some clusters presented protein products more hydrophobic than others, and that the stability could be compromised by this factor, as the thermo-stability decreased (see II and AI values). Despite the fact that SpA-I cluster is a rather homogeneous group, with only two isoforms, the observed increase on hydrophobicity and instability of its isoforms could be explained by the previously identified PRE (Fig. 5) that altered the protein characteristics by generating novel variations.

**Table 3:** Main characteristics of SpA alleles from *S. aureus* strain. Strains were sorted by Instability Index (II)

Strain	Length (aa)	pI	MW (Da)	Instability Index (II)	GRAVY	Aliphatic index (AI)
Mu50	330	5.01	36292.34	56.44	−1.48	48.91
Mu3	330	5.01	36292.34	56.44	−1.48	48.91
N315	330	5.01	36292.34	56.44	−1.48	48.91
10388	338	5.03	37162.27	56.71	−1.507	47.75
15532	338	5.03	37162.27	56.71	−1.507	47.75
16035	338	5.03	37162.27	56.71	−1.507	47.75
18341	338	5.03	37162.27	56.71	−1.507	47.75
10497	338	5.03	37162.27	56.71	−1.507	47.75
16125	338	5.03	37162.27	56.71	−1.507	47.75
18583	338	5.03	37162.27	56.71	−1.507	47.75
MSHR1132	338	4.96	36977.97	57.64	−1.478	49.2
133	404	4.89	44514.17	58.49	−1.466	50.12
ECTR2	388	5.03	42859.59	58.71	−1.416	52.45
JH1	388	5.03	42859.59	58.71	−1.416	52.45
JH9	388	5.03	42859.59	58.71	−1.416	52.45
NCTC8325	396	5.04	43856.62	58.84	−1.464	51.39
VC40	396	5.04	43856.62	58.84	−1.464	51.39
LGA251	395	4.92	43582.29	59.02	−1.437	52.51
JKD6159	372	4.9	41008.54	59.13	−1.339	54.7
FPR3757	388	5.03	42973.69	59.15	−1.432	52.45
COL	388	5.03	42973.69	59.15	−1.432	52.45
Newman	388	5.03	42973.69	59.15	−1.432	52.45
M1	380	5.01	42090.76	59.48	−1.4	53.55
08BA02176	380	5.02	42103.84	59.48	−1.4	53.82
04-02981	372	4.95	41136.71	59.59	−1.357	54.7
476	372	4.95	41193.76	59.82	−1.365	54.7
MW2	372	4.95	41193.76	59.82	−1.365	54.7
M013	364	5.03	40357.93	59.88	−1.338	55.91
252	396	5.05	43741.57	59.99	−1.448	51.39
TCH1516	372	4.95	41250.81	60.04	−1.373	54.7
71193	372	5.01	41277.97	60.46	−1.374	54.97
11819-97	364	5.09	40453.12	60.64	−1.35	55.91
JKD6008	306	5.08	33801.83	60.65	−1.358	55.33
ST398	364	4.94	40380.97	60.83	−1.337	56.18
TW20	306	5.01	33773.77	60.90	−1.355	55.33
T0131	298	5.06	32975.95	61.40	−1.325	56.81
TCH60	372	5.01	41206.89	61.50	−1.366	54.7

## Discussion

Given the role of SpA as crucial virulence-related effector enabling *S. aureus* to escape innate and adaptive immune responses, one can consider it a target for host specialization and clonal expansion through adaptive evolution. Indeed, *S. aureus* pathogenicity could be influenced by variations on *spA*. The observed incongruence between ML phylogenetic trees obtained from alignment of extracellular domains of *spA* locus and from MLST-concatenated alignment analysis (Fig. 2) was supported by mosaic gene patterns found in *spA* in which different gene segments exhibitting different evolutionary histories (Fig. 3). The influence of recombination and horizontal gene transfer events in the phylogenetic relationships among *S. aureus* strains were determined by a Neighbor-Net analysis. Several conflicting phylogenetic signals were observed throughout the network (Fig. 4), namely in cluster *spA*-II, suggesting that niche-specific selection pressures have been operating on this gene. In fact, it lead us to speculate that observed allelic diversity in *spA* could mirror fitness variations into virulence of those strains. Of the 38 *S. aureus* analyzed strains, 17 had at least one recombinant region and one of them presented two (Fig. 5). These findings reveal that the exchange of genetic material is apparently common in *S. aureus* and is in agreement with the report of the existence of hotspots in the core genome of this mostly clonal bacterium [80]. Our analysis revealed that PREs were not equally distributed through *spA* gene since predicted C domain was involved in all PREs and predicted B domain and Xr (a) region were implicated in four PREs, suggesting that these domains could represent recombination hotspots. These recombination events lead to the formation of mosaic genes potentially implicated on the generation of new biological properties. Another relevant result was the identification of PRE’s within and between CC’s, highlighting the importance of this mechanism on the generation of diversity, and concomitantly, on evolution of highly clonal *S. aureus*. Two different studies suggested that recombination in *S. aureus* was more likely to occur between closely related strains (i.e. within CCs) than between phylogenetic distant lineages (i.e. between CCs) [81, 82]. This would ultimately favor a divergence evolution between CC given limited gene flow observed between them. This model regards CCs as panmictic units (sexual species) rather than groups of clones as envisioned by the clonal model [83]. Surprisingly, our results did not confirm the pattern of higher recombination rate within CCs.

The low *dN*/*dS* ratios confirmed that purifying (negative) selection is operating in *spA* alleles and that variation are limited to those that do not cause a significant disadvantage. In tests used to detect departure from neutrality, values were non-significant suggesting that the null hypothesis of neutrality could not be rejected (Table 2). Therefore the pattern of variability observed in *spA* gene can be explained by the neutral process [57, 58, 72].

Our results confirm that most *spA* nucleotide polymorphisms resulted in amino acid changes. These data are not in accordance with other studies focused on the diversity of other *S. aureus* genes, namely, highly variable core adhesion (ADH) genes [84] and *aur* gene [85], where gene’s diversity was several-fold higher than that presented by MLST loci. Nevertheless, the abovementioned genes were under strong purifying selection when compared to the MLST genes [84, 85].

Pathogen fate could be drastically affected by amino acid substitutions on key virulence-factors. Indeed, amino acid variations were not randomly distributed in SpA and two groups of polymorphic sites were detected (Fig. 6), one encoding the immunoglobulin-binding domains D to C, and other the Xr (a) domain, as previously observed (Additional file 2: Figure S2). The abovementioned Ig domains of SpA (E-C) binds the Fcγ domain of immunoglobulin (Ig) and cross-links the Fab domain of V_H_3-type B cell receptors (IgM), playing an essential role in *S. aureus* escape from host immune system [9, 10, 14, 18, 19]. Accordingly, previous studies determined that amino acid substitutions in SpA at four key residues in each of the five Ig-binding promoted adaptive responses that protect hosts against recurrent infection [10]. Thus, the evolution of *spA* via frequent non-synonymous mutations could provide some *S. aureus* strains with increased fitness, reinforcing the importance of those domains.

From our analysis we have determined that not a single residue under positive selection was identified in SpA Ig binding domains and signal sequence, indicating that these SpA domains are under a strong negative constraint. However, several red and pink-colored sites were present in the partitions of SpA comprising the X region, representing positively selected codons with high statistical significance (Fig. 6). This domain is known to be related with SpA anchoring [86] so it is conceivable that evolution could act, namely by selecting duplications in this region, once a longer X region results in a better exposition of the Fc-binding region of protein A, or by altering the binding properties of the domain, in order to allow SpA a more easy access to the Fc of IgG [25, 26]. In sum, a selective advantage of those strains is expected by providing an increase on their fitness thereby facilitating colonization and/or contributing to the epidemic phenotype.

## Conclusion

Given the key role of SpA in *S. aureus* virulence we studied the mechanisms operating on its molecular evolution. The detection of positive selection operating on *spA* evolution was clear. Intragenic recombination, nonsynonymous mutations and duplication events are important strategies in the evolutionary adaptive process contributing to *spA* genetic plasticity. These events led to the formation of a mosaic gene composed by different segments with distinct evolutionary histories fostering novel biological properties. This could provide *S. aureus* strains with increased fitness, namely in the colonization of host surfaces or in Ig binding affinity, contributing to the epidemic phenotype by generating novel variations of SpA domains. Moreover, saving such allelic diversity/plasticity in nature imply that they represent selected adaptations.

## Abbreviations

BURP, Algorithm based upon repeat pattern; AI, Aliphatic Index; *k*, Average number of pairwise nucleotide differences; CC, Clonal complex; CA-MRSA, Community-associated MRSA; GRAVY, Grand Average of Hydropathy; HA-MRSA, Hospital-associated MRSA; Ig, Immunoglobulin; II, Instability Index; KEGG, Kyoto Encyclopedia of Genes and Genomes; ML, Maximum likelihood; LRT, Maximum Likelihood Ratio test; MRSA, Methicillin- Resistance *Staphylococcus aureus*; MSSA, Methicillin-Sensitive *Staphylococcus aureus*; MST, Minimum spanning tree; MLST, Multilocus sequence typing; NCBI, National Center for Biotechnology Information; *dN*, Nonsynonymous substitutions; π, Nucleotide diversity; *Hd*, Overall haplotype diversity; PREs, Potential Recombination Events; PFGE, Pulsed-field gel electrophoresis; SpA, staphylococcal protein A; *dS*, Synonymous substitutions

## Additional files

Additional file 1: Figure S1. Nucleotide polymorphism in *spA* of *S.aureus*. Sliding window plot of number of polymorphic sites (S) along *spA*, generated by using DnaSP. (PDF 248 kb) Additional file 2: Figure S2. Amino acid sequence polymorphism in SpA. Polymorphic amino acid residues are listed for each haplotype. Multiple sequence alignment of SpA was performed with ClustalW and visualized with Jalview. (PDF 362 kb) Additional file 3: Figure S3. Maximum likelihood phylogenetic trees of *S. aureus* strains (Table 1) from deduced amino acid sequences. Bootstrap support values (1,000 replicates) for nodes higher than 50 % are indicated next to the corresponding node. (PDF 96 kb) Additional file 4: Table S1. Potential recombinant events (PRE) identified with RDP3 from the alignment of *spA* from 38 *S. aureus* strains. The minimum number of independent recombination events (IREs) within each identified PRE was inferred by a minimum of three methods. (PDF 246 kb) Additional file 5: Table S2. Likelihood ratio tests of positive selection. (PDF 205 kb)
