# Supplementary material for: The recombination dynamics of Staphylococcus aureus inferred from spA gene
Source: BMC Microbiol. 2016 Jul 11;16:143. doi: 10.1186/s12866-016-0757-9 (PMC4940709; doi:10.1186/s12866-016-0757-9)
Supplement: Additional file 1: Figure S1. — Nucleotide polymorphism in spA of S.aureus. Sliding window plot of number of polymorphic sites (S) along spA, generated by using DnaSP. (PDF 248 kb) [file 12866_2016_757_MOESM1_ESM.pdf]

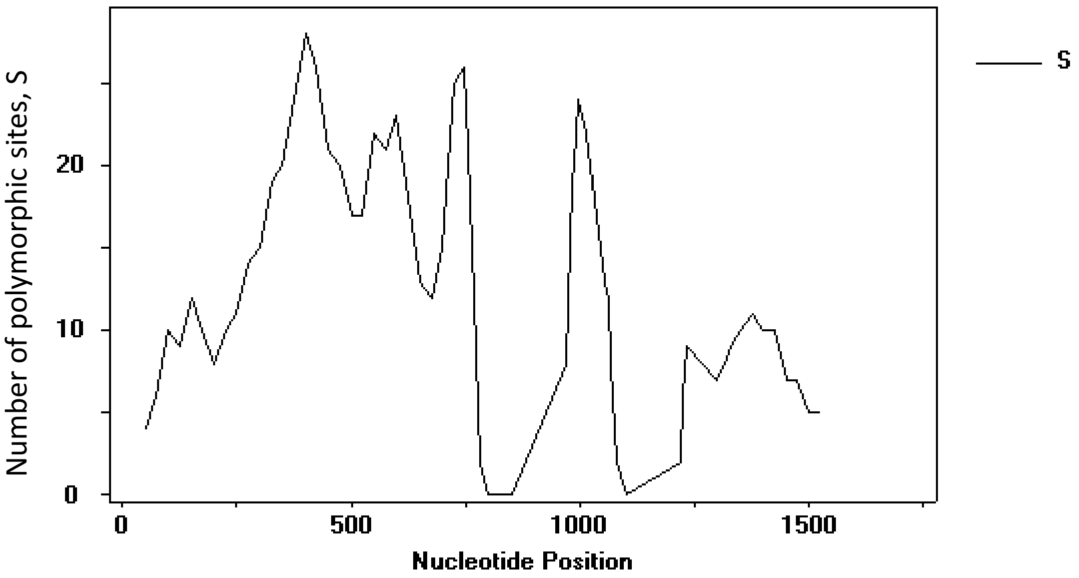

Figure S1. Nucleotide polymorphism in *spA* from *S. aureus* studied strains. Sliding window plot of number of polymorphic sites (S) along the *spA gene* generated by DnaSP ver. 5.10.00 with a window length of 100 bp and step size of 25 bp.
