# Supplementary figures and images for: The recombination dynamics of Staphylococcus aureus inferred from spA gene
Source: BMC Microbiol. 2016 Jul 11;16:143. doi: 10.1186/s12866-016-0757-9 (PMC4940709; doi:10.1186/s12866-016-0757-9)

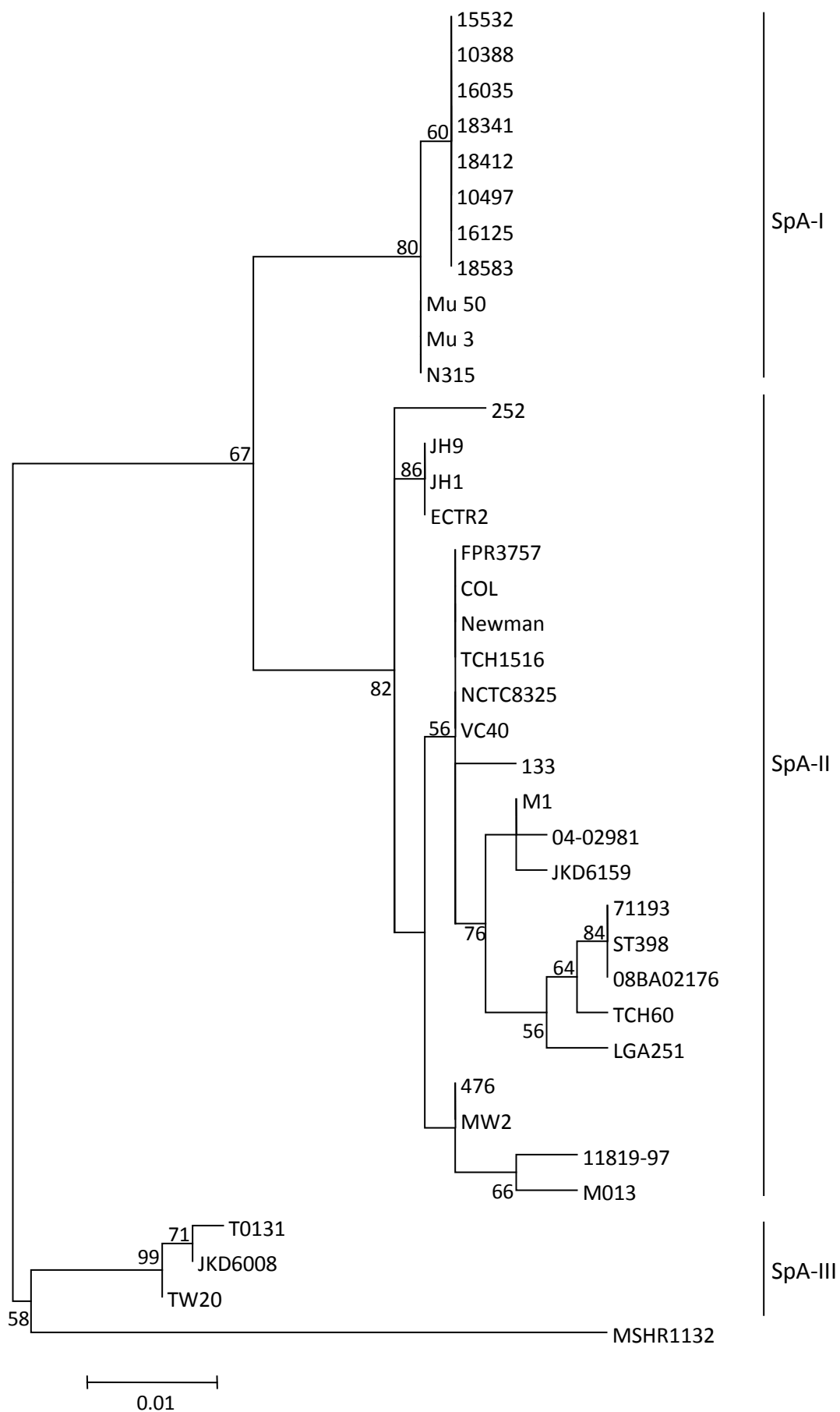

Supplement: Additional file 3: Figure S3. — Maximum likelihood phylogenetic trees of S. aureus strains (Table 1) from deduced amino acid sequences. Bootstrap support values (1,000 replicates) for nodes higher than 50 % are indicated next to the corresponding node. (PDF 96 kb) [file 12866_2016_757_MOESM3_ESM.pdf]
