# Supplementary material for: The recombination dynamics of Staphylococcus aureus inferred from spA gene
Source: BMC Microbiol. 2016 Jul 11;16:143. doi: 10.1186/s12866-016-0757-9 (PMC4940709; doi:10.1186/s12866-016-0757-9)
Supplement: Additional file 4: Table S1. — Potential recombinant events (PRE) identified with RDP3 from the alignment of spA from 38 S. aureus strains. The minimum number of independent recombination events (IREs) within each identified PRE was inferred by a minimum of three methods. (PDF 246 kb) [file 12866_2016_757_MOESM4_ESM.pdf]

Table S1. Potential recombinant events (PRE) identified with RDP3 from the alignment of *spA* from 38 *S. aureus* strains. The minimum number of independent recombination events (IREs) within each identified PRE was inferred by a minimum of three methods.

PRE	Breakpoints	IREs	*p*-Val	Recombination detection tests					
				RDP	GENECONV	MaxChi	Chimaera	SiScan	Bootscan
1	665-1211<br>357-884	11	$2.15x10^{-18}$	11	11	11	11	6	11
2	733-884	3	$1.07x10^{-3}$	3	3	3	3	3	3
3	98-1115	3	$2.14x10^{-18}$	3	3	3	3	3	3
4	888-1018	1	$2.61x10^{-7}$	1	-	1	-	-	1
5	1065-349	1	$7.72x10^{-5}$	-	-	1	-	1	1
