# Supplementary material for: The recombination dynamics of Staphylococcus aureus inferred from spA gene
Source: BMC Microbiol. 2016 Jul 11;16:143. doi: 10.1186/s12866-016-0757-9 (PMC4940709; doi:10.1186/s12866-016-0757-9)
Supplement: Additional file 5: Table S2. — Likelihood ratio tests of positive selection. (PDF 205 kb) [file 12866_2016_757_MOESM5_ESM.pdf]

Table S2- Likelihood ratio tests of positive selection.

Breaking points	Partitions (codons)	M8a	M8	2(M8-M8a)	*p*-value
All *spA*	1-1572 (1-525)	**-3798.55**	**-3765.55**	66	**<0.001**
1	1-327 (1-109)	-571.661	-571.648	0.026	no significante
2	328-648 (110-216)	-790.111	-790.11	0.002	no significante
3	649-915 (217-305)	-535,617	-535,620	-0,006	no significante
4	916-1572 (306-525)	**-1784.74**	**-1768**	**33.48**	**<0.001**

Column headings 8a7 and M8 denote the likelihood score. 2(M8-M7) denote test statistic and *p*-value the level of significance of the test statistic. In all *spA* and in the partition 916-1572 the Likelihood Ratio Tests indicate high probability of evolution under positive selection. *p*-values below 0.01 are marked in bold.
