# Supplementary material for: The recombination dynamics of Staphylococcus aureus inferred from spA gene
Source: BMC Microbiol. 2016 Jul 11;16:143. doi: 10.1186/s12866-016-0757-9 (PMC4940709; doi:10.1186/s12866-016-0757-9)
Supplement: Additional file 2: Figure S2. — Amino acid sequence polymorphism in SpA. Polymorphic amino acid residues are listed for each haplotype. Multiple sequence alignment of SpA was performed with ClustalW and visualized with Jalview. (PDF 362 kb) [file 12866_2016_757_MOESM2_ESM.pdf]

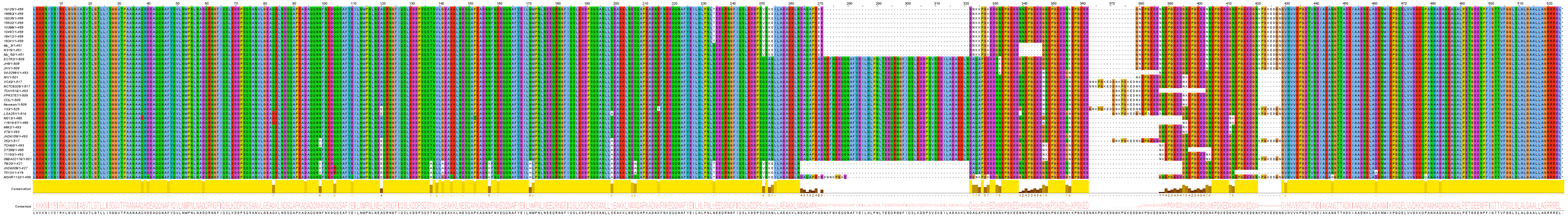
Conservation
Consensus
